# The evolution of hummock–depression micro‐topography in an alpine marshy wetland in Sanjiangyuan as inferred from vegetation and soil characteristics

**DOI:** 10.1002/ece3.7278

**Published:** 2021-03-25

**Authors:** Guiling Wu, Xilai Li, Jay Gao

**Affiliations:** ^1^ State Key Laboratory of Plateau Ecology and Agriculture Qinghai University Xining China; ^2^ College of Agriculture and Animal Husbandry Qinghai University Xining China; ^3^ School of Environment University of Auckland Auckland New Zealand

**Keywords:** alpine marsh wetlands, degradation, hummock–depression, micro‐topography, plant communities, soil properties

## Abstract

The hummock–depression micro‐topography characteristics of the alpine marshy wetland in Sanjiangyuan are indicative of wetland degradation and the process by which healthy wetlands are transformed into flat grasslands. The aim of the present study was to examine changes in plant community structure and soil characteristics in a hummock–depression micro‐topography along a degradation gradient. We observed that: (a) the height and cover of dominant hydrophytes decreased gradually with an increase in degradation severity, leading to replacement by xerophytes; (b) with the transition from healthy to degraded wetlands, hummocks became sparser, shorter, and broader and became merged with nearby depressions; water reserves in the depressions shifted from perennial to seasonal, until they dried out completely; and (c) soil moisture content, porosity, hardness, and organic matter gradually decreased by 30.61%, 19.06%, 37.04%, and 73.27%, respectively, in hummocks and by 33.25%, 8.19%, 47.72%, and 76.79%, respectively, in depressions. Soil bulk density, soil electrical conductivity, and soil dry weight increased by 31%, 83.33%, and 105.44%, respectively, in hummocks, but by only 11.93%, 7.14%, and 97.72%, respectively, in depressions. The results show that hummock soils in healthy wetlands have strong water absorption properties, through which plant roots can penetrate easily. Wetland degradation reduces the water absorption capacity of hummock soil and soil saturation capacity of depressions, thus enhancing soil erosion potential and susceptibility to external factors. Soil moisture is a key environmental factor influencing wetland degradation, and grazing accelerates the process. Based on the changes observed in hummock morphology, vegetation, and soil properties along a degradation gradient, a conceptual model is proposed to illustrate the process of gradual degradation of marshy wetlands from healthy to transitional wetlands and finally to a degenerated state. Thus, our research provides insights into the degradation process of the alpine marshy wetland ecosystem in Sanjiangyuan.

## Graphical abstract

This study aims to examine the changes in plant community structure and soil characteristics in the hummock–depression micro‐topography along the degradation gradient. Thus, based on the changes in hummock morphology, vegetation, and soil properties, a conceptual model is proposed to clearly illustrate the process of the gradual degradation of the marshy wetland from healthy to transitional, and finally to the degenerated state.

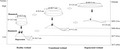

## 1 INTRODUCTION

**FIGURE 1:**
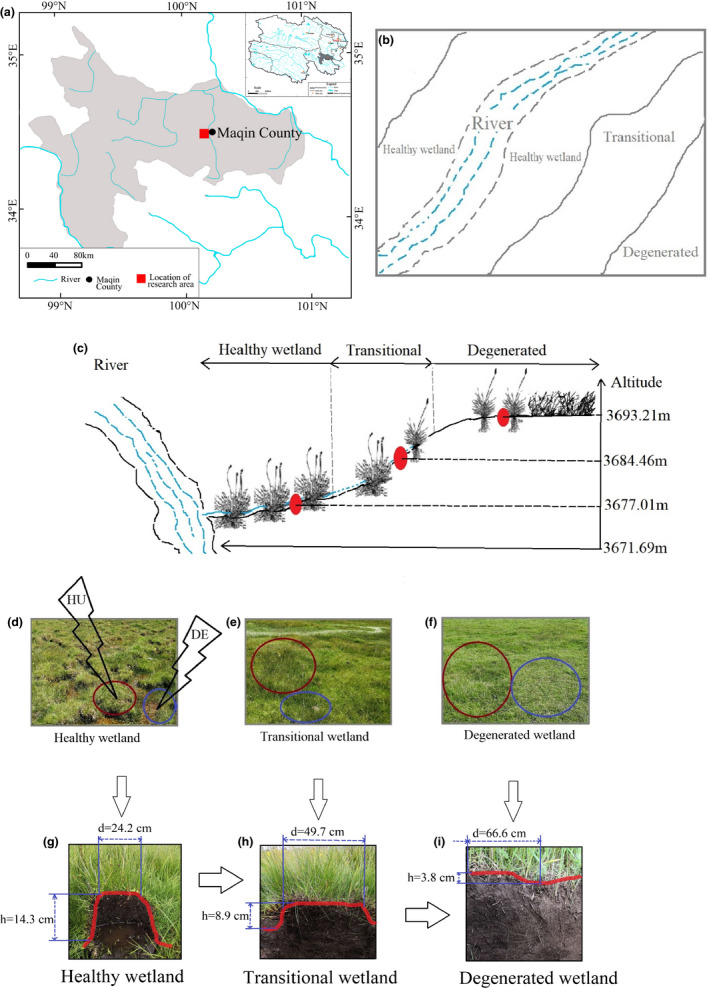
Map of the study site and sampling site distribution

The alpine marshy wetland in Sanjiangyuan in western China is a critical water conservation and biodiversity reservoir. The unique, pristine, and fragile wetland is also a valuable pasture resource. Numerous low‐temperature hummocks are widely distributed in the wetland (Biasi et al., 2005; Oddi et al., 2019; Pintaldi et al., 2016; Zhao et al., 2020). In addition, it is dotted with poorly drained depressions, with characteristics typical of *Kobresia tibetica* meadows (Li et al., 2016; Lin et al., 2019). The alternation between dry hummocks and wet depressions creates a micro‐topography that is unique to the alpine marshy wetland (Gao et al., 2013; Li et al., 2016; Lin et al., 2019) and contrasts strongly with the local natural environment (Pintaldi et al., 2016; Shen et al., 2006; Zhao et al., 2020). The spatial juxtaposition of hummocks and depressions of varying sizes in close proximity creates an uneven surface and a micro‐topography (Figure 1d–f) that is highly sensitive and vulnerable to external factors (Nungesser, 2003). The meadows have been subjected to a series of ecological challenges in recent years, such as drought and increased desertification, due to the combined impacts of climate change and anthropogenic activities (Chen et al., 2002; Li et al., 2016; Lin et al., 2019; Yu et al., 2010). In some areas, short‐rooted plants have died, leading to reduced biodiversity (Chen et al., 2020; Ma et al., 2010; Wang, Li, et al., 2008). Consequently, the spatial patterns and structure of the wetland ecosystem have changed considerably. Global change has seriously altered the micro‐topography and disrupted the ecological equilibrium in the wetland ecosystem (Chen et al., 2002; Li et al., 2016).

It is essential to study the micro‐topography of wetland ecosystems since it influences soil spatial heterogeneity through the physical and chemical properties, which, in turn, significantly influence meadow plant growth and survival (Deák et al., 2015; Oddi et al., 2019; Rose & Malanson, 2012). The geometry and spatial distribution of the hummock‐depressions complex influence material circulation and energy flows in marshes, in addition to soil nutrient and particle characteristics (Diamond et al., 2019; Pintaldi et al., 2016; Zhao et al., 2020). Furthermore, they mediate the feedback between community structure and ecosystem functionality and enhance spatial variability in soil resources. Change in hummock–depression micro‐topography with an increase in degradation severity serves as a key indicator of the mechanisms underlying the evolution of marshy wetland ecosystems (Wang et al., 1997). Therefore, investigating the spatial distribution patterns and the characteristics of hummocks and depressions in such alpine marshy wetlands at different degradation stages could provide a valuable reference and theoretical basis for their conservation.

Environmental heterogeneity exists across different spatial scales. For alpine wetlands, the scale is micro (e.g., <1 m^2^) for some resources such as water, nutrients, and light (Diamond et al., 2019; Vivian‐Smith, 1997), and the hummock–depression micro‐topography in the alpine marshy wetlands in Sanjiangyuan in western China is on a miniature scale of this type. Despite the existence of several theories to explain the formation of hummock–depression micro‐topography (Diamond et al., 2019; Edgar, 1998; Li et al., 2018; Tallis & Livett, 1994; Wang et al., 1997), no researcher has compared the physical and chemical properties of hummocks and depressions in the alpine marshy wetlands quantitatively, even though some authors have reported interactions between the development of alpine marshy wetlands and moisture, climate change, and litter decomposition (Wang et al., 2007; Zhang et al., 2016; Zhao et al., 2020). Therefore, it remains unclear how hummock–depression complexes vary under different levels of degradation severity, and how such variations influence vegetation and soil interactions between hummocks and depressions along degradation gradients. In‐depth knowledge of such dynamics would enhance our understanding of the process of alpine marshy meadow degradation. Differences in soil physicochemical and plant characteristics, and plant succession in hummocks and depressions under different degradation states could reveal the mechanisms underlying the degradation of alpine marshy wetlands.

The present study aimed to determine the sizes and shapes of hummocks and depressions in an alpine marshy wetland in Sanjiangyuan in western China and to explore relationships between the sizes and shapes of hummocks and depressions and the plant community and soil properties along the degradation gradient to reveal plant succession characteristics. The specific objectives were: (a) to assess hummock and depression physical properties along a degradation gradient; (b) to examine the plant community structure and soil properties in relation to micro‐topography; (c) to determine the coupling relationship between hummock dimensions and soil and plant characteristics; and (d) to propose a conceptual model for elucidating degradation from alpine marshes to grasslands.

## 2 MATERIALS AND METHODS

### 2.1 Study species

Four indicator plants were selected for study, namely *Kobresia tibetica*, *Kobresia pygmaea*, *Carex scabrirostris*, and *Carex muliensis*. *Kobresia tibetica* is a coloniser of hummocks, while *Kobresia pygmaea* is a typical indicator species in alpine meadows (Dand et al., 2014; Wang, Cao, et al., 2008). The degradation status of alpine meadows is usually evaluated based on the presence or disappearance of the above species (Dand et al., 2014; Lin et al., 2019; Miehe et al., 2019; Wang, Cao, et al., 2008). *Carex scabrirostris* and *Carex muliensis* are typical species in alpine marshy depressions. They have considerable biomass in hypoxic environments (Zhao, 1998); therefore, the surface water status of the depressions can be assessed based on their productivity.

### 2.2 Study area

The study area is located in the southern Dawu Town, Maqin County, Guoluo Prefecture of Qinghai Province (34°28′–34°46′N, 100°12′–100°22′E) with an altitude of 3671–3693 m a.s.l. (Figure 1c). The area lies in a mountainous valley of the Upper Yellow River in Sanjiangyuan (Figure 1a). It has a cold plateau climate with no distinct seasonality excluding general cold and warm seasons. The cold season generally lasts 7–8 months, and is windy, bitterly cold, and snowy, while the warm season is relatively short (4–5 months) and largely humid and cool. The area receives high solar radiation, with the annual sunshine hours totaling 2,576 hr. Annual temperature averages only −2.6°C, with a small range. Annual average precipitation is 513 mm. There is no completely frost‐free period throughout the year, and the growing season is limited to May‐September. Alpine marshy wetlands, which are used predominantly for livestock grazing all year round, are distributed widely in the study area. Winter pastures are grazed approximately 240 days a year from September to April, while summer pastures are grazed for approximately 120 days per annum from May to August. The study site was selected because the marshy alpine wetland has been degraded quite severely since the 1990s. Decreased water resources in the wetlands and degraded wetland vegetation in the area threaten the sustainability of the ecological environment and the livelihoods of resident herders (Li et al., 2018; Liu et al., 2019). Therefore, urgent interventions are required to prevent further degradation of the alpine marshy wetland ecosystems and to restore the degraded wetland where possible.

### 2.3 Field sampling

Field sampling was conducted in August 2019. Survey plots were set up in a large number of hummocks and depressions in the wetland used as winter pasture (Figure 1d–f) radially along the degradation gradient (Table 1). The plots were set up in three gentle (<10°) zones (healthy, transitional, and degenerated) centered along a river (Figure 1b). The healthy zone represents the original state of the marshy wetland that has not demonstrated any signs of change. The degenerated zone is so dry that it is virtually an alpine meadow, whereas the transitional zone lies between these two extremes. Triplicate 1 m by 1 m plots were distributed randomly within each zone to ensure their representativeness. All the plots were radial from the interior to the exterior in a circular band. They were distributed over a wide spatial area to capture the temporal succession of vegetation (Zhou et al., 2005); however, the effect of grazing was not taken into account since all the plots were located within the winter pasture. Additional considerations were given to hummock morphology and quantity, water volume in a depression, *Kobresia tibetica* dominance, and vegetation cover. The key properties of vegetation communities within each plot were surveyed, including plant species, grass height, and vegetation cover.

**TABLE 1:** Characteristics of sampling sites in the three degradation zones

Degradation gradient	Total cover (%)	Species richness (mean ± *SD*)	Dominant species	Major features
Healthy	95–100	13 ± 3	Hummocks: *Kobresia tibetica,* *Kobresia capillifolia* Depressions: *Carex muliensis,* *Carex scabrirostris*	The area has numerous elliptical hummocks and butterfly‐shaped depressions dominated by *Kobresia tibetica*. The depressions mostly have permanent surface water accumulation, and the drainage is generally poor. The grass is neat and monotonous dark green in appearance.
Transitional	93–98	17 ± 3	Hummocks: *Kobresia tibetica,* *Kobresia humilis* Depressions: *Carex muliensis,* *Carex scabrirostris*	Most of the area is still dominated by *Kobresia tibetica*; however, at relatively lower abundance. Hummocks have decreased significantly when compared to the healthy state, and the volume of the seasonal water in depressions is obviously reduced, with alternating dry and wet states and seasonal water accumulation.
Degenerated	91–95	20 ± 2	Hummocks: *Kobresia humilis,* *Kobresia pygmaea* Depressions: *Carex muliensis,* *Carex scabrirostris*	Dominated by *Kobresia humilis*, with numerous grasses of various types, and dry soil. Depression surfaces are relatively flat and without water. A few hummocks or low hummocks are almost connected with depressions and have evolved into alpine meadows.

To evaluate the micro‐topography, similar sampling activities were carried out in two other marshy alpine wetlands spaced more than 5 km apart. Three identical 5 × 5 m plots were set up in each degradation zone. The number of hummocks within each plot was counted, and their diameter and height were measured, in addition to water depth in the depressions. Soil samples were collected from each plot by removing surface litter. To ensure the randomness and representativeness of the collected samples, the midpoints of the diagonal lines of the 5 × 5 m plots were used as the central sampling point, along which four samples were collected at the same spatial intervals. Vegetation and soil samples were collected from both hummocks and depressions within three 1 × 1 m sub‐plots (three replicates) after surface litter had been removed from each sub‐plot.

### 2.4 Soil property measurement

Soil samples were collected near the surface (0–10 cm) with a ring knife with an internal diameter of 3.5 cm. In total, 18 soil samples were collected from each sub‐plot (54 samples in total). After mixing, the soil samples were stored in pre‐tagged bags, tightly closed, and then transported to the laboratory. After air‐drying, the soil samples, from which animal and plant residues had been removed, were ground and sieved to 0.15–2 mm aggregate sizes for use in soil biophysical and chemical property analyses. Soil organic matter was analyzed using the dichromate oxidation method (Kalembasa & Jenkinson, 1973), and soil hardness was measured using a soil compactness meter (SC900 digital display type, Spectrum). Soil moisture, conductivity, and pH value were measured on‐site using a portable soil moisture temperature conductivity meter (Spectrum TDR 350).

Soil bulk density was determined using the ring knife method; a ring knife with an internal diameter of 3.5 cm containing the soil sample is dried at 105°C to a constant weight and then used to calculate soil bulk density.

(1) $$\rho_{b}=\frac{m_{1}‐m_{2}}{V}\times 100$$

where $\rho_{b}$ is soil bulk density (g/cm^3^); *m*_1_ is soil dry weight and the mass of ring knife (g); *m*_2_ is the mass of the ring knife (g); and *V* is the volume of the ring knife (cm^3^).

(2) $$P=\left(1‐\frac{\rho_{b}}{\rho }\right)\times 100\%$$

where *P* is soil porosity (%); ρ is soil density, the usual density value = 2.65 g/cm^3^.

### 2.5 Data analysis

The collected soil chemical and physical data were analyzed using Excel 2018 (Microsoft Corp.). IBM SPSS Statistics 23.0 (IBM Corp.) was used to perform generalized linear modeling analysis and to perform one‐way analysis of variance, and, subsequently, the least significant difference post‐test, to compare the different characteristics of plant communities and soil characteristics under different degradation states (healthy wetland, transitional wetland, and degenerated wetland). Origin 2018 (OriginLab Corp.) was used to illustrate the relationship between hummock morphology and soil properties.

## 3 RESULTS

### 3.1 Plant community structure

**FIGURE 2:**
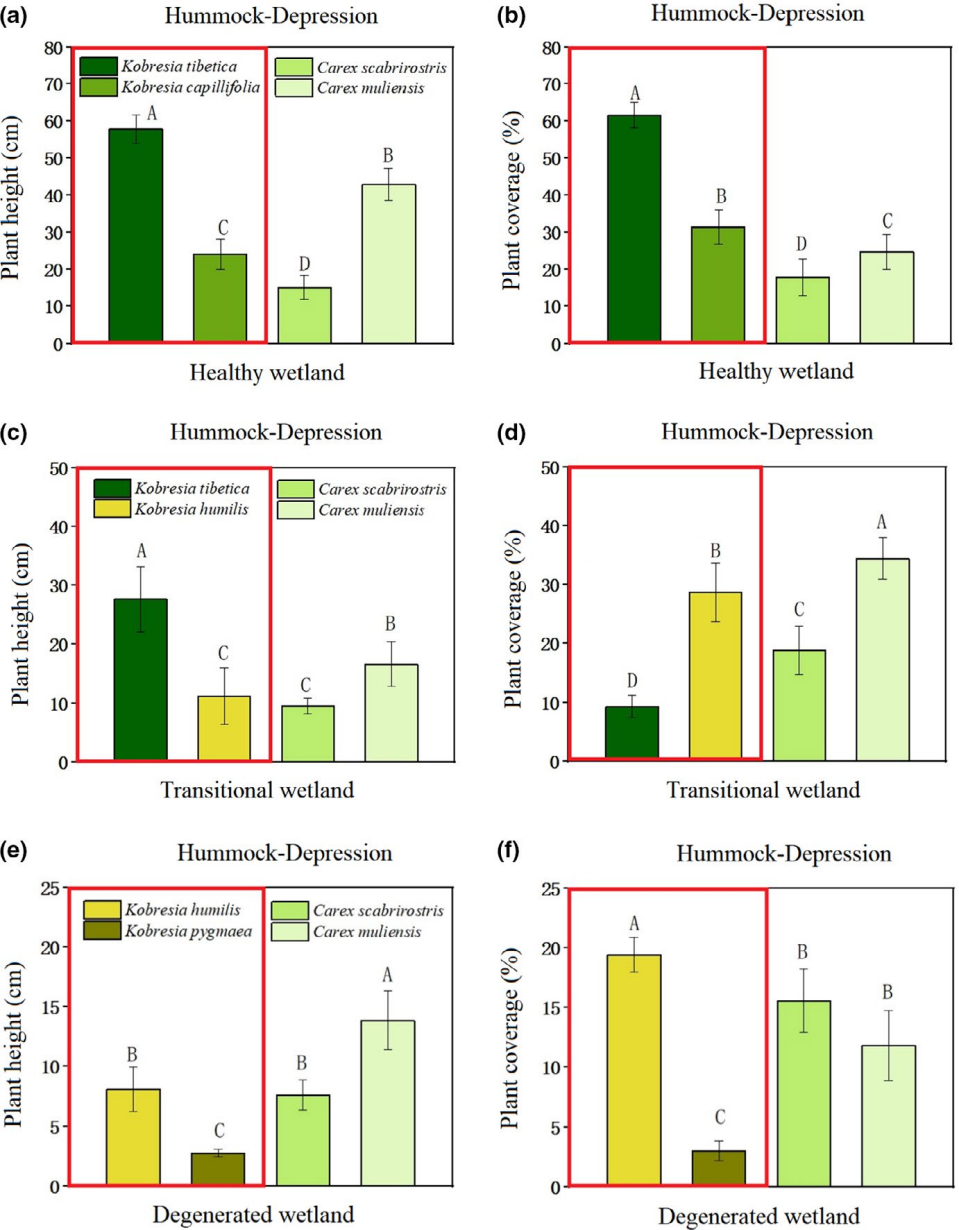
Hummock–depression indicative plant height and cover under different degradation states. Note: Capital letters indicate plant height or plant coverage. Different letters indicate significant differences between treatments (*p* < .05). Similar letters indicate no significant difference, among which A > B > C > D

*Kobresia tibetica* and *Kobresia capillifolia* dominated the hummocks; however, *Carex scabrirostris* and *Carex muliensis* were dominant in the depressions in the healthy wetland. Plant height and plant coverage were significantly different (*p* < .05) among the four species (Figure 2a,b). *Kobresia tibetica* requires relatively abundant water resources and rich organic matter to develop; therefore, it thrives in healthy alpine marshy wetlands. The height of the dominant species *Kobresia tibetica* decreased by 52.19% from the healthy wetland to the transitional zone, and its cover decreased by 84.91% on hummocks; the height of the dominant species, *Carex scabrirostris* and *Carex muliensis*, decreased by 36.68% and 61.22%, respectively, in depression, while their plant cover increased by 5.6% and 39.33% (Figure 2). In the degenerated zone, *Kobresia tibetica* disappeared completely, and *Kobresia humilis* occupied the dominant niche, with its height and plant cover on hummocks decreasing by 27.55% and 32.32%, respectively. The height of *Carex scabrirostris* and *Carex muliensis* in the depressions decreased by 20.04% and 16.61%, respectively, and their plant cover decreased by 17.44% and 65.64%, respectively; in contrast, species diversity increased.

In the transitional zone, *Kobresia tibetica* was still the dominant species in hummocks; however, its height and coverage were significantly lower than in healthy wetlands. The associated species, *Kobresia humilis*, had replaced *Kobresia capillifolia* in the zone. Although *Carex scabrirostris* and *Carex muliensis* were still present, their height and coverage were significantly lower than in the depressions in healthy wetlands (Figure 2c,d), because *Kobresia capillifolia* prefers to grow on round, hilly, moist, and thick soils that are rich in organic matter (Hu et al., 2019). In addition to the hummocks in healthy wetlands being relatively tall, their soil layers were thicker, the soil moisture contents were higher, and the organic matter contents richer (Table 2), which are all conducive for *Kobresia capillifolia* growth. In the degenerated zone, hummocks were dominated by *Kobresia humilis* and *Kobresia pygmaea*, and the height and cover of *Carex scabrirostris* and *Carex muliensis* in depressions were lower than those in the transitional zone (Figure 2e,f).

**TABLE 2:** Variability in soil physical and chemical properties

Level	Healthy wetland			Transitional wetland			Degenerated wetland		
	HU (±*SD*)	DE (±*SD*)	|ΔCV|	HU (±*SD*)	DE (±*SD*)	|ΔCV|	HU (±*SD*)	DE (±*SD*)	|ΔCV|
SMC (%)	67.43 (±1.04)	68.76 (±0.50)	0.81	64.85 (±1.16)	62.81 (±2.81)	2.68	46.79 (±2.89)	45.90 (±3.11)	0.60
SP (%)	62.42 (±0.89)	58.71 (±1.74)	3.7	68.76 (±7.50)	59.13 (±0.75)	9.64	50.52 (±1.99)	53.90 (±1.71)	0.77
SBD (g/cm^3^)	1.00 (±0.02)	1.09 (±0.05)	2.59	0.83 (±0.20)	1.08 (±0.02)	22.24	1.31 (±0.05)	1.22 (±0.05)	0.27
SH (kg/cm^2^)	1,031.68 (±131.63)	1,227.20 (±74.51)	6.69	926.44 (±76.05)	1,124.24 (±48.90)	3.86	649.56 (±15.64)	641.52 (±13.07)	0.37
SEC (ms/cm)	0.24 (±0.04)	0.42 (±0.05)	4.90	0.14 (±0.05)	0.43 (±0.07)	19.89	0.44 (±0.04)	0.45 (±0.04)	0.20
SDW(g)	32.74 (±0.29)	34.24 (±2.84)	7.42	28.94 (±5.96)	40.54 (±2.78)	13.72	67.26 (±13.39)	67.70 (±12.91)	0.83
pH	6.83 (±0.31)	7.09 (±0.37)	0.60	6.75 (±0.55)	6.66 (±0.29)	3.86	6.99 (±0.55)	7.00 (±0.53)	0.21
SOM (g/kg)	410.85 (±23.86)	446.54 (±35.52)	2.15	362.91 (±22.66)	383.27 (±27.48)	2.45	109.80 (±22.57)	103.63 (±21.01)	0.28

Note

HU represents hummocks; DE represents depressions. |ΔCV| represents the absolute value of the difference between the coefficients of variation of HU and DE, which illustrates the spatial heterogeneity of micro‐topography under different degrees of degradation. *SD* represents standard deviation. (SMC, soil moisture content; SP, soil porosity; SBD, soil bulk density; SH, soil hardness; SEC, soil electrical conductivity; SDW, soil dry weight; pH, soil pH; SOM; soil organic matter, *n* = 240).

### 3.2 Soil properties

From the healthy zone to the transitional zone, soil moisture, hardness, and organic matter, and pH, and bulk density decreased by 3.83%, 10.20%, 11.67%, 1.17%, and 17.00%, respectively, in hummocks, and by 8.65%, 8.39%, 14.17%, 6.06%, and 0.92% in depressions, respectively. In hummocks and depressions, porosity increased by 10.16% and 0.72%, respectively; however, electrical conductivity and dry weight decreased by 41.67% and 11.61%, respectively, in hummocks and increased by 2.38% and 18.40%, respectively, in the depressions (Table 2). Excluding soil moisture, soil pH and bulk density decreased quite differently between hummocks and depressions, while all other properties declined at similar degrees. Soil porosity increased more significantly in hummocks than in depressions. However, the greatest difference between hummocks and depressions was observed in soil conductivity and soil dry weight, which decreased in hummocks, but increased in depressions. At this stage, the decreases in soil moisture, organic matter and pH in depressions were slightly higher than in hummocks, and the decreases in soil hardness and soil bulk density in hummocks were more significant than those in depressions.

However, from the healthy zone to the degenerated zone, soil porosity and soil hardness decreased to a large degree because healthy wetlands have high soil porosity and high water‐holding capacity ( Thompson, 2020; Zhou et al., 2019). Soil hardness in hummocks and depressions decreased by more than 37%, while soil bulk density, soil electrical conductivity, and soil dry weight increased by 31%, 83.33%, and 105.44%, respectively, in hummocks, notably higher than those in depressions (11.93%, 7.14%, and 97.72%, respectively) (Table 2). The greatest difference between the two was the change in soil conductivity, which increased significantly in hummocks, but only slightly in depressions, indicating that there were more salt ions in the soil during degradation. In general, soil moisture and organic matter decreased by the same degree in hummocks and depressions from the healthy zone to the degenerated zone.

From the transitional zone to the degenerated zone, soil moisture, hardness, porosity, and organic matter decreased by 27.85%, 29.89%, 26.53%, and 69.74%, respectively, in hummocks and by 26.92%, 42.94%, 8.84%, and 72.96%, respectively, in depressions. Soil pH, bulk density, electrical conductivity, and soil dry weight in hummocks increased by 3.56%, 57.83%, 214.28%, and 132.41%, respectively, in hummocks but by 5.1%, 12.96%, 4.65%, and 67.00%, respectively, in the depressions. Therefore, soil properties in hummocks are more vulnerable to degradation than in depressions. Notably, at different stages of degradation, soil properties in hummocks and depressions change at different rates. For instance, soil hardness decreased by 10.14% from healthy wetlands to the transitional zone, but by 29.89% from the transitional zone to the degenerated zone (Table 2). Soil water content, organic matter, and hardness in both hummocks and depressions decreased with an increase in degradation and was especially rapid from the transitional zone to the degenerated zone.

The variability in soil quality indicators can be appreciated based on coefficients of variation (CV) (CV ≤ 10%‐weak; 10% ≤ CV ≤ 100%‐moderate; and CV ≥ 100%‐strong) (Abdi, 2010; Bu et al., 2014). The absolute difference in CV between hummocks and depressions is used to illustrate the magnitude of change in micro‐topography. The physical and chemical properties of hummocks and depressions clearly changed along the degradation gradient (Table 2). The difference between hummocks and depressions in the transitional and healthy zones was much greater than that in the degenerated zone. The difference was the greatest in the transitional zone, but the least in the degenerated zone. Therefore, the transitional zone is a wet‐dry nexus area where soil properties fluctuate considerably with a change in water reserves. The least difference observed in the degenerated zone confirmed that, as the alpine wetlands were degraded, the difference in height between hummocks and depressions decreased, as did the difference in soil properties.

### 3.3 Relationship between hummocks and vegetation and soil properties

**FIGURE 3:**
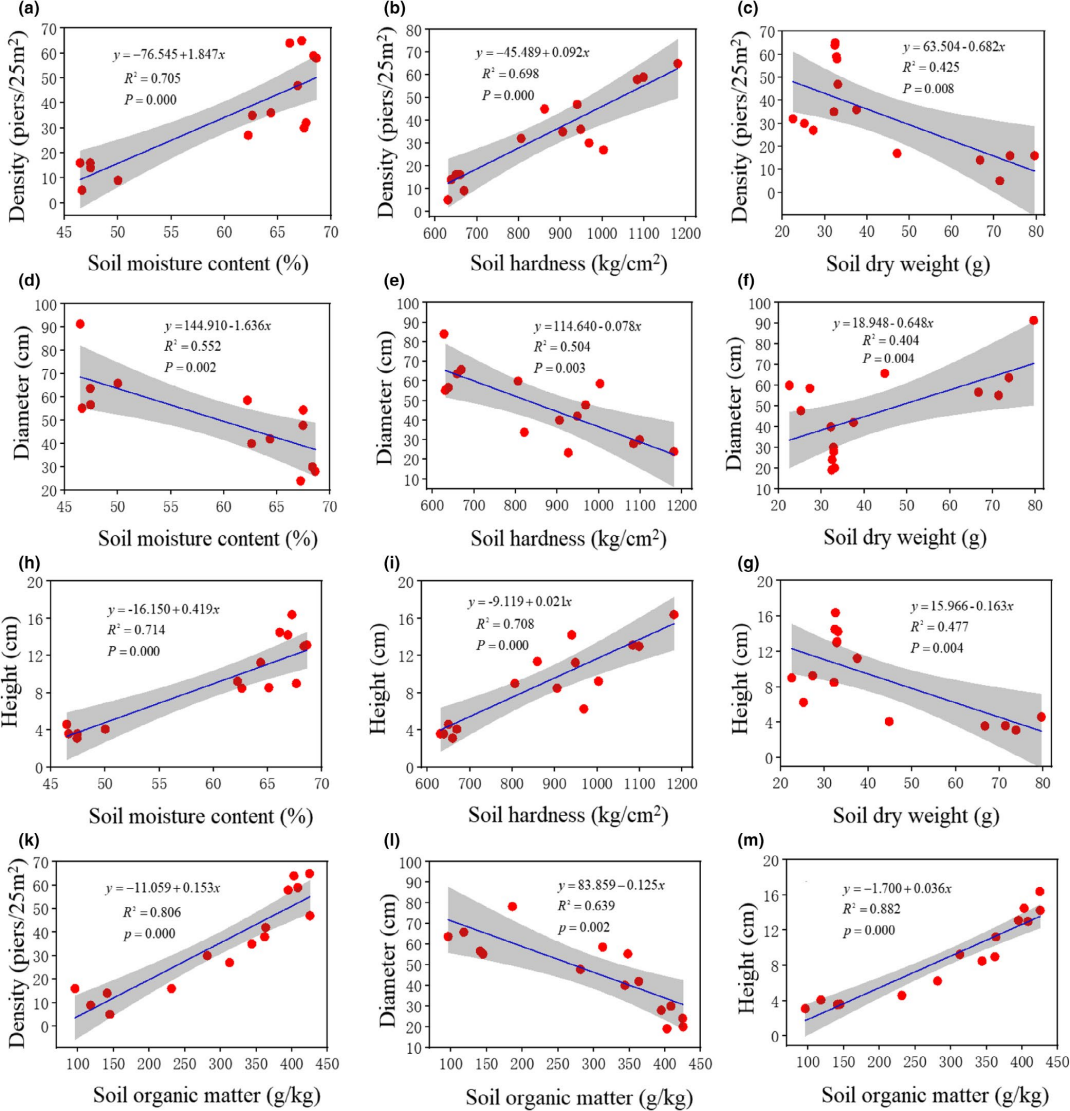
Regression relationships between hummock characteristics and soil properties. Note: Density represents number of hummocks in a 25‐m^2^ area; Diameter represents the diameter at the top of the hummock; Height represents the distance from the top of the hummock to the depression

**FIGURE 4:**
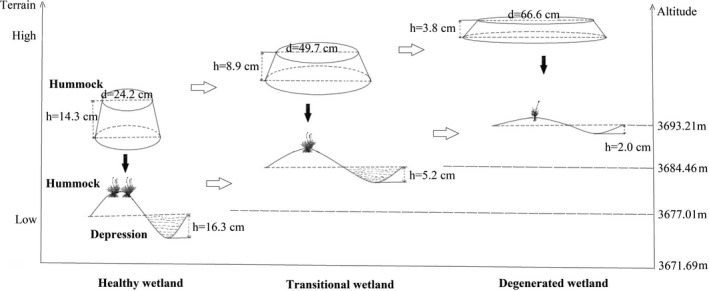
Alpine marshy wetland degradation and change in hummock–depression micro‐topography and structure

The hummocks in the three degradation zones had significantly different density, diameter, height, and nearest neighbor distance (Tables 3, 4). Furthermore, hummocks in healthy wetlands had significantly smaller diameters than their counterparts in the other two zones. Hummock diameter more than doubled from 24.2 cm in healthy wetlands to 49.7 cm in the transitional zone. However, it increased by only 34% in the degenerated zone (Table 3; Figure 4). Therefore, the initial stages of degradation are most influential for changes in diameter. Hummock diameter was positively correlated with degradation severity. Hummock height dropped from 14.3 cm in healthy wetlands to 8.9 cm in the transitional zone, a 37.76% decrease (Table 3). The decrease increased to 57.3% in the degenerated zone (Figure 4). In addition, the nearest neighbor distance among hummocks increased from 63.37 cm in healthy wetlands to 91.60 cm in the transitional zone, representing a 44.55% increase, and increased further to 57.14% in the degenerated zone (Figure 4). Therefore, the more degraded the wetlands, the shorter the hummocks were. There was a good coupling relationship between hummock shape and size, and vegetation and soil properties (Table 5 and Figure 3). Hummock density decreased along the degradation gradient, halved from 60 piers/25 m^2^ in healthy wetlands to 31 piers/25 m^2^ in the transitional zone, to only 13 piers/25 m^2^ in the degenerated zone (Table 3). Therefore, the quantity was notably lower under more severe levels of degradation, and the nearest neighbor distance between hummocks was continually increasing.

**TABLE 3:** Hummock–depression properties under three degradation zones

Level	Healthy wetland				Transitional wetland				Degenerated wetland			
	Mean	*SD*	Max	Min	Mean	*SD*	Max	Min	Mean	*SD*	Max	Min
Density (piers/25 m^2^)	60 a	3	64	56	31 b	2	34	29	13 c	1	14	11
Diameter (cm)	24.2 c	3.8	30.0	19.0	49.7 b	4.3	60.0	40.0	66.6 a	8.6	91.4	55.2
Height (cm)	14.3 a	1.4	16.4	13.0	8.9 b	1.8	11.2	6.2	3.8 c	0.6	4.6	3.1
Nearest Neighbor Distance (cm)	63.37 c	14.90	110	40	91.60 b	34.21	180	47	143.94 a	40.54	275	76
Water depth (cm)	−16.3 c	−2.9	−20.0	−11.5	−5.2 b	−0.6	−6.2	−4.6	0 a	0	0	0

Note

The letters a, b, and c represent differences between healthy, transitional, and degenerated wetlands (*p* < .01), where a > b > c. Density refers to the number of hummocks in 25 m^2^ area; Diameter refers to the diameter at the top of the hummock; Height refers to the distance from the top of the hummock to the bottom depression; Water depth refers to the height of the water in the depression; Nearest Neighbor Distance refers to the distance between two adjacent hummocks; *n* = 280.

**TABLE 4:** Pearson correlation between hummock density, and diameter, height, and nearest neighbor distance and *Kobresia tibetica* cover, height, and aboveground biomass

	Density	Diameter	Height	Distance	COV	HE	AGB
COV	0.754**	−0.603*	0.793**	−0.871**	1.000	0.925**	0.686**
HE	0.676**	−0.698**	0.725**	−0.856**	0.925**	1.000	0.667**
AGB	0.821**	−0.844**	0.832**	−0.743**	0.686**	0.667**	1.000

Note

Density represents to the number of hummocks in a 25 m^2^ area; Diameter represents the diameter of the top of the hummock; Height represents the distance from the top of the hummock to the bottom of the depression; Nearest Neighbor Distance (Nearest Neighbor Distance) represents the distance between two adjacent hummocks; COV refers to *Kobresia tibetica* cover; HE represents *Kobresia tibetica* height; AGB represents aboveground biomass of *Kobresia tibetica* (**p < .01, *p < .05); *n* = 90.

**TABLE 5:** Relationship between hummocks and *Kobresia tibetica* population attributes

Project (*y*)	HU (*x*)	Relational model	*R* ^2^	*F*	*p*	*n*
COV	Density	y=‐22.524+1.307x	0.819	40.619	.000	15
	Diameter	y=101.950‐1.897x	0.854	52.496	.000	15
	Height	y=‐36.034+6.234x	0.769	29.748	.000	15
	Distance	y=128.888‐1.359x	0.759	40.956	.000	15
HE	Density	y=5.413+0.810x	0.705	21.533	.001	15
	Diameter	y=78.489‐1.066x	0.605	13.773	.005	15
	Height	y=2.167+3.392x	0.510	9.374	.014	15
	Distance	y=104.917‐0.922x	0.733	35.678	.000	15
AGB	Density	y=‐21.153+6.409x	0.649	26.833	.000	15
	Diameter	y=506.060‐6.580x	0.712	32.157	.000	15
	Height	y=‐60.364+28.819x	0.692	29.171	.000	15
	Distance	y=665.659‐6.630x	0.552	16.008	.002	15

Note

*y* represents the cover (COV), height (HE), and aboveground biomass (AGB) of *Kobresia tibetica*; *x* represents the density, diameter, and height of hummocks. Density refers to the number of hummocks in a 25 m^2^ area; Diameter refers to the diameter of the top of the hummock; Height refers to the distance from the top of the hummock to the depression; Distance refers to the distance between adjacent hummocks; COV represents *Kobresia tibetica* plant cover; HE represents height of *Kobresia tibetica*; AGB represents aboveground biomass of *Kobresia tibetica*.

Water depth in the depressions could only be compared between healthy wetland and the transitional zone since water had dried up in the degenerated zone. Water levels in the depressions in healthy wetlands were significantly higher than those in the transitional zone (*p* < .01) (Table 3). The average height differences between hummocks and depressions were 30.6 cm in healthy wetland, 14.1 cm in the transitional zone, and 5.8 cm in the degenerated zone. As the wetland degrades, the height difference decreased gradually. In the degenerated zone, the height difference was almost nonexistent (Table 3, Figure 4).

The cover, height, and aboveground biomass of *Kobresia tibetica* bore close linear relationships with the densities, diameters, heights, and nearest neighbor distances of hummocks (all *R* ^2^ ≥ 0.50), suggesting alpine meadow degradation can be predicted based on *Kobresia tibetica* population characteristics (Tables 4, 5). Compared to the vegetation characteristics of non‐degraded *Kobresia tibetica*, it is possible to predict the degradation status of alpine wetlands, which could serve as the scientific evidence for the proper management and restoration of degraded wetlands.

Furthermore, hummock density, diameter, and height all had close linear correlations with soil water content, soil hardness, soil dry weight, and soil organic matter. As illustrated in Figure 3, dense hummocks were correlated with high soil water content, high soil hardness, high organic matter, but low soil mass. The taller the hummocks (*R* ^2^ = 0.714, *p* < .01), the smaller their diameter (*R* ^2^ = 0.552, *p* < .01), the denser they were (*R* ^2^ = 0.705, *p* < .01), and the higher their soil moisture content. In addition, the greater the hardness of the hummock soil, the taller the hummocks (*R* ^2^ = 0.708, *p* < .01), the lower the hummock diameter (*R* ^2^ = 0.504, *p* < .01), and the denser the hummocks (*R* ^2^ = 0.698, *p* < .01). Furthermore, the taller the hummocks (*R* ^2^ = 0.806, *p* < .01), the higher their soil organic matter, the lower their diameter (*R* ^2^ = 0.639, *p* < .01), and the greater their density (*R* ^2^ = 0.882, *p* < .01). Overall, as the water disappeared from the depressions, the difference in hummock and depression height shrank. A reduction in micro‐topographic surface roughness was not conducive for the retention of soil nutrients and moisture, and the soil structure changes, which, in turn, minimized the differences in properties between hummocks and depressions.

## 4 DISCUSSION

### 4.1 Micro‐topography versus vegetation properties

Hummock diameter changed more than hummock height along the degradation gradient, which could be explained by three processes. First, climate change decreased hummock water content and shrank their volume. Global climate change, especially in the form of warmer temperatures at high latitudes, has led to the disappearance of permafrost in hummocks, melting of seasonal permafrost, thickening of active layers, and xerophyte vegetation encroachment, in addition to the inhibition of *Kobresia tibetica* growth by seasonal waterlogging (Åkerman & Johansson, 2008; Lemke et al., 2007; Pintaldi et al., 2016). According to Grab (2005), Hsueh (2013) and Krüger et al. (2014), permafrost formation leads to the uplift of peat layers and formation of hummocks. Consequently, the temperature rise has potentially accelerated the process of degradation of peat, and the disappearance of permafrost has caused the collapse of hummocks (Marushchak et al., 2011; Repo et al., 2009). Such processes also affect hydrology, vegetation composition, carbon balance, and other biogeochemical processes of peat during freezing and thawing (Bäckstrand et al., 2010; Christensen et al., 2004; Malmer et al., 2005; Olefeldt & Roulet, 2012). Therefore, climate change has led to severe drought and degradation of alpine marshy wetlands (Gao et al., 2012). Second, the continuous trampling of alpine marshy wetlands by livestock has flattened and expanded the hummocks, decreasing the height difference between hummocks and depressions (Figures 1 and 4). Third, as degradation intensifies, the soil becomes less resilient and can be eroded easily by external forces, which decreases hummock height (Wang, Cao, et al., 2008; Xie et al., 2012; Yang et al., 2010), as revealed by the positive linear relationship between soil hardness and hummock height and the negative linear relationship between hummock height and hummock diameter. By the degenerated stage, hummocks were bigger and shorter (Figure 1 and Table 3). When hummock heights decreased to a certain level, they were mostly integrated with the depressions, and the hummocks became inconspicuous. Eventually, adjoining hummocks coalesced to form larger ones, resulting in a decrease in the density of hummocks, and an increase in the nearest neighbor distances between hummocks.

There are complex and intricate interactions among the observed changes in micro‐topography, vegetation and soil properties. The hummock–depression micro‐topography controls the local soil moisture distribution (Edgar, 1998; Wang et al., 1997), which, in turn, leads to significant differences in vegetation composition between hummocks and depressions. *Carex muliensis* height decreased significantly, while its cover increased significantly because of a 68.1% reduction in the surface water in the depressions, which hinders the survival of hydrophytes and vulnerable species (Wang et al., 2007). Wetland degradation caused *Kobresia tibetica* in the hummocks of healthy and transitional wetlands to gradually decrease. The apparent decline in the surface water resulted in the emergence of many low‐growing *Carex scabrirostris* and *Carex muliensis* individuals in the depressions. As the soil moisture decreased and the wetland dried up, the hydrophytes *Kobresia tibetica* and *Kobresia capillifolia* exhibited stress and formed xerophytic structures within their tissues (Wang, Cao, et al., 2008). They were succeeded gradually by *Kobresia humilis* and *Kobresia pygmaea*. The warmer and drier climate created an environment suitable for *Carex scabrirostris* and *Carex muliensis* to grow in low‐lying and swampy depressions. As the wetlands dried up and degraded, their height and cover declined. *Kobresia tibetica* and *Kobresia capillifolia* either decreased their biomass or disappeared gradually due to stronger inter‐species competition caused by the gradual invasion of *Kobresia humilis* (Lin et al., 2013; Wang, Cao, et al., 2008). Furthermore, the area occupied by *Kobresia humilis* expanded as a result of decreasing *Kobresia tibetica* and *Kobresia capillifolia* populations. As degradation worsened, *Kobresia pygmaea* invaded and replaced *Kobresia tibetica*. Such gradual succession of the plant community along the degradation gradient illustrates the differential responses of the plant species to the habitat (van Andel et al., 1993).

In a previous study, the plant cover of an initially dominant species, *Primula nutans*, was more sensitive to changing climate than its height (Shen et al., 2006). Minor differences in soil moisture can lead to significant differences in seed germination in wetland communities (Vivian‐Smith, 1997), leading to significant differences in their floral diversity. Therefore, healthy and degenerated wetland habitats are very different. Compared to healthy wetlands, degenerated wetlands have soil moisture and nutrient levels that are 30% and 73% lower, respectively. The decomposition rates of soil nutrients in healthy wetlands are much lower than those in degenerated areas (Gao et al., 2012). Healthy wetlands are characterized by resilient hummocks that can resist environmental erosion and maintain high levels of nutrients in the soil. The close correlation between hummock and vegetation properties is explained as follows. With the gradual degradation of wetlands, the hummocks with the increased surface area can absorb more solar radiation and prevent soil moisture oversaturation, in turn improving photosynthesis efficiency (Mark, 1994; Tarnocai & Zoltai, 1978), which may influence wetland vegetation succession. In degraded wetlands, *Cyperaceae* plants are replaced by *dicotyledonous* weeds, which have high photosynthetic efficiency and low water use efficiency (Li et al., 2012). Therefore, the hummocks tilted and collapsed into the depressions due to external interference (Nijp et al., 2019; Pintaldi et al., 2016), which resulted in the formation of larger hummocks between adjacent hummocks, a significant reduction in height difference between hummocks and depressions, and the gradual establishment of a uniform habitat.

### 4.2 Micro‐topography versus soil properties

Changes in micro‐topography have implications for small‐scale changes in soil texture (Grab, 1997; Grab, 2005), bulk density (Dee & Ahn, 2012), and conversion and maintenance of soil nutrients (Biasi et al., 2005). Soil electrical conductivity and dry weight first decreased and then increased in the hummocks, reaching levels greater than the gradual increases in the depressions. In addition, soil bulk density and pH in hummocks and depressions decreased and then increased. Thus, in the alternating dry and wet transitional zone, the bulk density of hummocks and depressions is the smallest. Compared to healthy wetlands, the soil peat content decreased (Ma et al., 2020; Pintaldi et al., 2016) and the soil organic matter decomposition capacity increased (Ali et al., 2017; Pintaldi et al., 2016). The surface soil is composed of decomposed and semi‐decomposed grass roots and organic matter, with a lower mineral content (Wang et al., 2020), so soil bulk density is smaller. The decreased differences in height between the hummocks and the depressions homogenized the microhabitat and affected the adaptability and diversity of plant species (Ma et al., 2020; Smith et al., 2012). Compared to changes in plant communities in hummocks, soil properties are less sensitive to degradation. Overall, vegetation is more sensitive to the changing external environments than soil (Cao et al., 2007), especially moisture availability. Distinct changes occurred to vegetation even at the early stages of degradation. For instance, *Kobresia humilis* individuals increased continuously in the transitional zone, replacing *Kobresia tibetica* and *Kobresia capillifolia* in their niches. Along the degradation gradient, soil bulk density in hummocks and depressions varied greatly; soil bulk density in hummocks increased more than that in depressions because of large numbers of *Kobresia tibetica* roots and decreased organic matter decomposition in healthy wetland (Table 1). In addition, the mineral content is low; therefore, soil bulk density is low, and the soil has a loose structure with high porosity. On the contrary, in the degenerated zone, there are numerous grass roots that decompose easily, soil organic matter is reduced, and mineral content and soil bulk density are high.

Increasing soil conductivity inhibits biological activity in the soil and impairs normal plant growth, reduces plant productivity, and affects organic matter input in the soil and soil carbon emissions (Ali et al., 2017). Excluding the different trends in electrical conductivity and soil dry weight in the first stage, hummocks and depressions exhibited similar changes in all other soil properties. Therefore, hummocks and depressions change with wetland degradation.

### 4.3 Marshy wetland degradation process

Wetland degradation leads to plant community succession, which, in turn, alters soil physical and chemical properties (Diamond et al., 2019; Gao & Li, 2016; Li et al., 2012). Besides, wetland degradation alters soil texture and soil organic matter and nutrient consumption, which influences microbial activities and communities (Abril & Bucher, 1999; Biasi et al., 2005), and, in turn, soil mineralization (Pintaldi et al., 2016). In the course of degradation, the rate of change and succession in plant properties is much more rapid than that in soil properties (Wang et al., 2020). This trend suggests that degradation is a continuous process that can be discerned based on plant succession, which is virtually a continuous process (van Andel et al., 1993; Vivian‐Smith, 1997). In addition, degradation can occur over a broad temporal scale, depending on the pace of the external environment change (Yang, 2019; Pintaldi et al., 2016). Therefore, the process cannot be studied effectively via chronological sampling. Such a challenge can be circumvented by sampling soil and vegetation along the degradation gradient. Therefore, the three zones selected capture the temporal sequence of degradation (Peng et al., 2012; Wang et al., 2006; Zhou et al., 2005). Although spatial sampling as a proxy for a chronological process could generate some errors, the present study strictly controlled the selection of plots and the demarcation of sampling zones. For example, a typical natural marshy wetland dominated by *Kobresia tibetica*, the difference in altitude (3,671–3,677 m), the surrounding environment, slope position, and slope direction of the plots all ensured similarity across plots, and that the disturbances in the course of succession were consistent. The similarity in succession conditions ensures the reliability of inferences made with regard to temporal change based on spatial change (Peng et al., 2012; Zhou et al., 2005).

**FIGURE 5:**
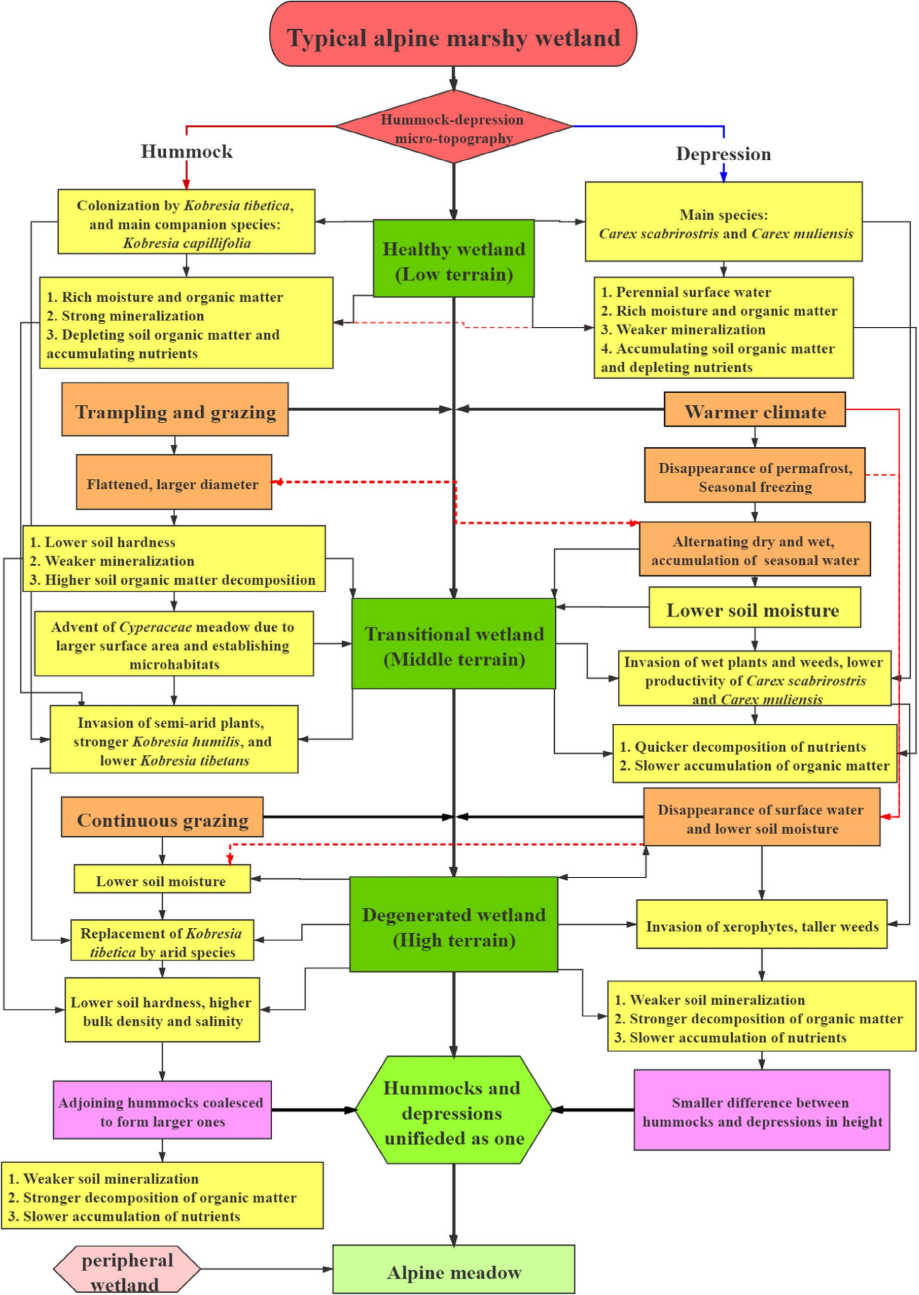
Conceptual model of alpine marshy wetland degradation

The sampling sites of the present study are located over a broad range of elevations (Figure 4). Although they have been subjected to the same degree of climate change, they respond differently due to their varying topographic settings. The healthy wetlands occur at the lowest elevation, 3,671.69–3,677.01 m, where moisture converges. The abundant water reserves in the area make them highly resilient to external changes. Therefore, a large number of well‐developed hummocks and depressions with sufficient water was largely intact. The transitional zone, which is at a higher elevation (3,677.01–3,684.46 m) than that of the healthy wetland zone, is characterized by dry and wet alternating hummocks and depressions. Considering the vegetation and soil properties of the zone, it must have evolved from a healthy marshy wetland. However, how long the process took remains unknown, as the answer depends on the pace of climate change. In contrast, the degenerated zone is located at the highest elevation (3,684.46–3,693.21 m), which is the most susceptible to the external factors, as water is diverted to low‐lying areas without sufficient recharge from higher grounds. There, the hummocks are the widest, but the shortest. In addition, their lower density is attributed to the coalescence with adjoining hummocks, which is a continuation of the gradation process from the transitional zone (Figures 4 and 5). The continuous change in vegetation composition and properties, as well as the delayed change in soil properties, further demonstrate the evolution from the transitional state. Again, it remains unknown how long it takes to shift from the transitional state to the degenerated state or whether the degenerated state can revert to the former, healthier state. The future trend of change of the degenerated wetland also remains unknown. Further research is required to investigate the outcomes if the environment is degraded further and if it would lead to the formation of a “black soil beach” meadow (Li, 2002; Li & Huang, 1996).

The transitional and degenerated zones require different strategies of restoration in zones where the wetland has transformed into an alpine meadow. Interpretation of satellite images revealed that alpine marshy wetlands in Maduo County near the study site shrank by nearly 17.34% from 1990 to 2001, but increased from 2001 to 2013 due to increased rainfall and warmer temperatures (Gao et al., 2016; Li et al., 2016). In addition, the area of alpine wetlands converted into nonwetlands decreased from 87.3% to 28.7%. Therefore, slightly degraded wetlands and dried wetlands are interchangeable, and the latter can be restored to wetlands naturally as climate changes and rainfall increases. However, wetlands that have been degraded into alpine meadows cannot be recovered naturally (Cao & Long, 2009). Therefore, the degeneration stage is the most critical in wetland restoration and reversibility (Ballantine & Schneider, 2009). Although there are a few artificial methods of recovering degraded wetlands (Ballantine & Schneider, 2009; Bruland & Richardson, 2006), their effectiveness remains questionable. Further research should be conducted to develop effective restoration strategies.

## 5 CONCLUSIONS

The micro‐topography of hummocks and depressions in the alpine marshy wetlands of the Sanjiangyuan profoundly influenced soil and vegetation properties. Grazing and climate change have led to severe drying and degradation of alpine marshy wetlands. Soil moisture is a critical environmental factor influencing wetland degradation that is accelerated by grazing. Wetland degradation altered vegetation community structure in hummocks. Such changes were closely associated with hummock characteristics, which, in turn, affected wetland vegetation, and soil moisture and nutrients. Based on the changes, it can be inferred that wetland degradation begins at the highest elevation in hummocks, even though hummocks and depressions exhibit similar trends of change. Therefore, hummock–depression micro‐topography degradation is a gradual process resulting in a transition from native wetlands to arid wetlands, and eventually to alpine meadows. In the process of wetland degradation, the resilient hummocks can resist environmental erosion and stabilize soil nutrient contents at high levels. Thus, the results of the present study could enhance our overall understanding of the process of degradation of the alpine marshy wetland ecosystem in Sanjiangyuan.

## CONFLICT OF INTEREST

None declared.

## AUTHOR CONTRIBUTION

**Guiling Wu:** Conceptualization (lead); Data curation (lead); Formal analysis (equal); Funding acquisition (equal); Investigation (equal); Methodology (equal); Project administration (equal); Writing‐original draft (lead); Writing‐review & editing (equal). **Xilai Li:** Conceptualization (supporting); Data curation (supporting); Formal analysis (supporting); Funding acquisition (supporting); Investigation (supporting); Methodology (supporting); Project administration (supporting); Resources (supporting); Supervision (supporting). **Jay Gao:** Conceptualization (supporting); Data curation (supporting); Formal analysis (supporting); Investigation (supporting); Methodology (supporting); Writing‐original draft (equal); Writing‐review & editing (equal).

## Data Availability

Basic data supporting the findings of this study are available within the manuscript. Data on plant characteristics will be published in the Dryad Data Repository data repository after publication, https://doi.org/10.5061/dryad.98sf7m0hp.
